# Supplementary figures and images for: Salt stress induces endoplasmic reticulum stress-responsive genes in a grapevine rootstock
Source: PLoS One. 2020 Jul 30;15(7):e0236424. doi: 10.1371/journal.pone.0236424 (PMC7392237; doi:10.1371/journal.pone.0236424)

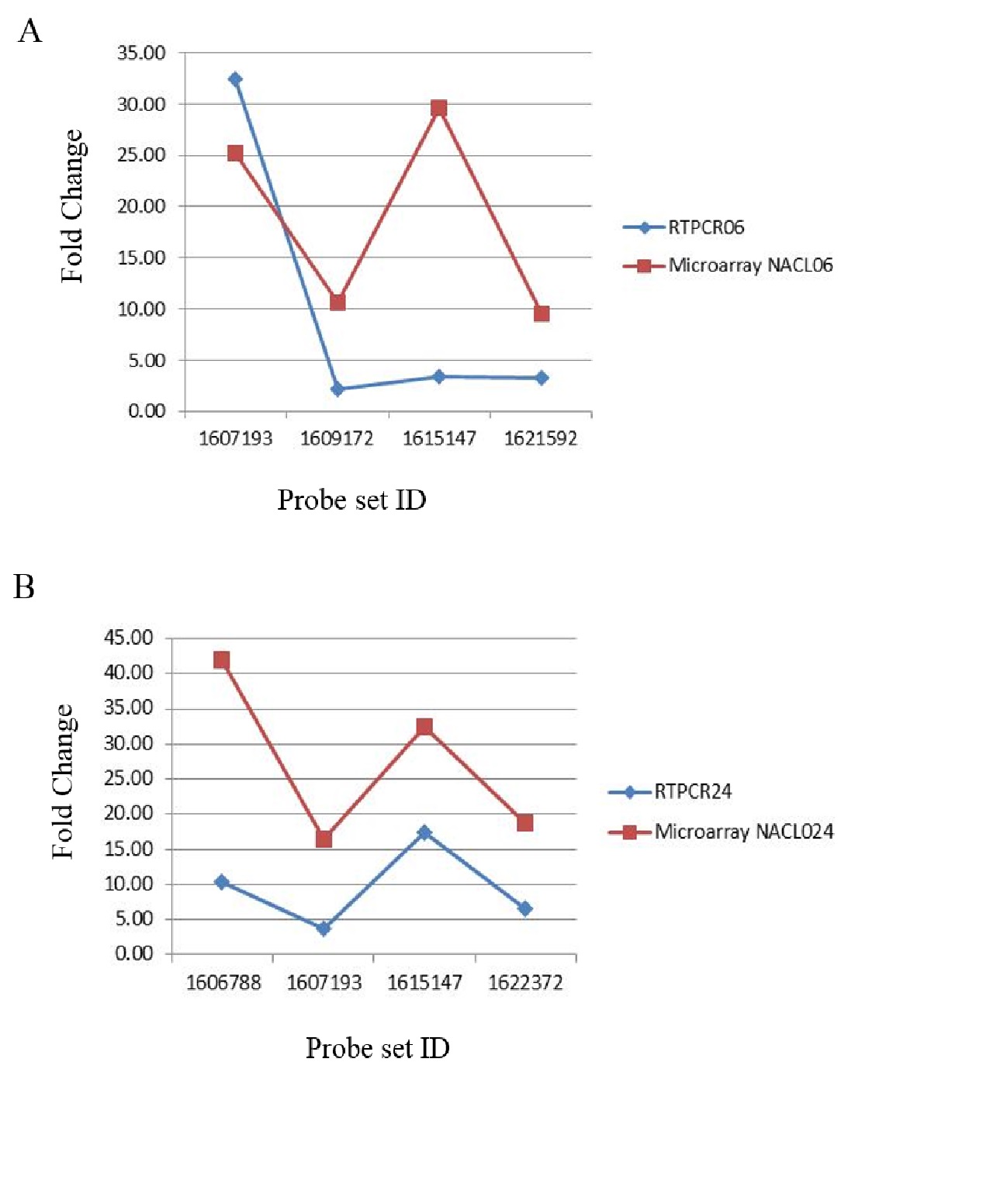

Supplement: S1 Fig — (JPG) [file pone.0236424.s001.jpg]
