# Supplementary material for: Salt stress induces endoplasmic reticulum stress-responsive genes in a grapevine rootstock
Source: PLoS One. 2020 Jul 30;15(7):e0236424. doi: 10.1371/journal.pone.0236424 (PMC7392237; doi:10.1371/journal.pone.0236424)
Supplement: Attachment — Submitted filename: PONE-D-20-03767_SH.docx [file pone.0236424.s006.docx]

PONE-D-20-03767- Review Scott Hayes

Salt stress induces endoplasmic reticulum stress-responsive genes in a grapevine rootstock

The authors present their work comparing the transcriptomes of salt and Tm treated grapevine. This data-set has the potential to inform future work and may prove a valuable contribution to the field. On the whole, the author’s discussion of their results is well measured and appropriate for the level of evidence they provide. I do however feel that the manuscript could be improved if the authors were to provide additional information on the experimental set-up. In addition, communication of their results could be improved through changes in the way they present their data.

One major point (and a pet peeve of mine!) is that at several occasions in the methods, readers are referred to the methods of other manuscripts. Not only does this waste a lot of the interested reader’s time, it may result in misunderstandings about how the work was actually performed. For example, the authors state that they performed RNA extraction from leaf samples as described in reference 52. As reference 52 is a comparison of 15 different RNA extraction methods, it is presently unclear which method they are referring to! In the interest of improving the reproducibility of these results, please include the full methods for this manuscript.

It would be useful if the figures and their associated legends contained more information.

**“Fig 1. Stem water potential of leaves treated with salt and ER stresses. Data are means of eight measurements”**

- Which leaves?

- How old were the plants?

- How were they grown?

- At what time did the treatment start?

Some of this information is in the method but having this basic information in the figure legend would help the reader to interpret the results. Additionally, the authors represent the mean of 8 measurements and so it would be good to include error bars in the figure to demonstrate the variance in the data. The authors also imply that they have performed statistical analysis of this data, but it is not clear from the figure legend what this entails.

**“Fig 2. Venn diagram for transcripts regulated at 6 h (A) and 24 h (B) by salt and ER stresses. Light green or blue represent transcripts with increased or decreased expression under salt stress, respectively. Yellow or green represent transcripts with increased or decreased expression under TM treatment”**

- Personally I found this figure legend quite difficult to understand at first. I believe that the authors are comparing transcript abundance between the control conditions and NaCl or TM treatments but this is not stated in the legend.

- In my opinion, the clarity of the legend could be improved by separating out the clauses in the sentences. For example, “Microarrays were performed on control, NaCl and TM treated plants at 6 h (A) and 24 h (B). Green circles show transcripts that were at a higher abundance in NaCl than the control, and blue circles show transcripts that were at a lower abundance in NaCl than the control. Red circles show…..”

- Also I would like to request that authors use a different colour scheme, as to me, the difference between green, light green and blue is not very clear.

**“Fig 4. Gene Ontology classification of salt-stress responsive genes. (A) indicates ’biological process’ and (B) indicates ‘molecular functions’ (C) indicates ‘cellular component’ for transcripts with increased or decreased expression under salt stress. Each bar represents the number of differentially expressed transcripts in each category”**
This figure contains so much data that it becomes difficult to see what it represents. It may be worth investigating other methods to display this data, such as a heat map for example.

**“Figure 5. Fig 5. Expression analysis of chosen genes by qRT-PCR. Total RNA was isolated from leaves of grapevine rootstock treated with NaCl (A, B) or tunicamycin (TM) (C, D) for 2. 6. 24. and 48. hours indicated times. The bars represent the relative intensity of labelled qRT-PCR products from three independent biological replicates. eIF4α was used as an internal control.”**

- It is not clear to me what the difference is between A & B, and C & D.

- Could the authors clarify what is meant by biological repeat? In many of their samples the variation between repeats is so small that error bars are not visible. In my experience, such small error bars are often seen for technical repeats (showing pipetting error from a single sample) than biological repeats (showing the difference in expression between completely separate experiments).

- The data also appears to have been normalised relative to Ctr. If so, this should be stated.

- If the purpose of this figure is to compare the expression of genes between TM and NaCl treatments it may be more useful to incorporate this information in a single graph for each gene. At the very least, the separate graphs could be rearranged so that they occupy the same order between the two treatments (e.g. both the top left figures showing stilbene synthase, and to their left dehydrin etc).

Subheadings such as “Effects of salt and ER stress on cell wall and fatty acid composition” and “Effects of salt and ER stress on chromatin structure” should be changed. Something like “The effect of salt and TM on the expression of genes that are involved in cell wall modification” is much less likely to give the reader the impression that cell wall composition or chromatin structure were experimentally evaluated in this manuscript. There are several examples of these headings in the discussion that could be improved.

I am also interested in some technical aspect of the experimental set-up. The authors submerge the roots of their pants in liquid media containing NaCl or Tm and then take samples of the leaves at 6 and 24hs thereafter. My question is: is there is any data on the speed at which Tm travels through the plant? The relatively low number of genes affected in the leaves is surprising, given the huge effect that Tm has on stem water potential.

An alternative hypothesis may be that Tm and NaCl both damage the root (leading to the drop in water potential and possible long-distance stress signal to the leaves). NaCl may then additionally travel to the leaves to elicit the large change in gene expression seen in this tissue. Would the authors care to comment on this?

Minor comments:

Page 2:

- “salt stress can be avoided by using salt-tolerant grape rootstocks obtained from wild Vitis

Species”. Please add a reference here.

Page 3:

-Some aspects of the introduction could contain more information to enhance the readability of the MS. For example, the salt sensitivity of slp and bZip17 mutants is discussed but it is assumed that the reader already knows the function of these genes. Similarly, instead of “IRE1 protein is highly conserved in eukaryotes including plants”, something along the lines of “IRE1 is an ER-localised transmembrane kinase that is well conserved in eukaryotes” may be more informative to readers from outside the field of ER stress. As is features heavily in the presented work, it would also be useful to state in the introduction what tunicamycin is and give a brief description of how it works.

Methods:

“As revealed by preliminary experiments, a quantity of 400 mM salt was sufficient for

decreasing stem water potentials while not harming the plants exposed to salt stress for the indicated times”. Please provide more details here.

Page 7:

“The expression of 107 genes changed in response to ER stress induced by TM (Fig 2A and 2B).”

This statement is incorrect as there are several overlapping genes between the two time points, meaning that the number of genes affected is lower than 107. The same holds for similar statements made regarding salt-regulated genes on page 8.

Page 18:

“…the transcript expression of an auxin binding protein ABP19a-like, which is involved in auxin signal perception,”. Could the authors please provide a reference here.
