# Supplementary material for: Salt stress induces endoplasmic reticulum stress-responsive genes in a grapevine rootstock
Source: PLoS One. 2020 Jul 30;15(7):e0236424. doi: 10.1371/journal.pone.0236424 (PMC7392237; doi:10.1371/journal.pone.0236424)
Supplement: Attachment — Submitted filename: Response to reviewers.docx [file pone.0236424.s008.docx]

PONE-D-20-03767R1
Salt stress induces endoplasmic reticulum stress-responsive genes in a grapevine rootstock
PLOS ONE

Dear Dr. Ali ERGÜL,

Thank you for submitting your manuscript to PLOS ONE. After careful consideration, we feel that it has merit but does not fully meet PLOS ONE’s publication criteria as it currently stands. Therefore, we invite you to submit a revised version of the manuscript that addresses the points raised during the review process.

One of the reviewers has requested for some minor formatting corrections. The authors need to address those points before getting final acceptance.

Please submit your revised manuscript by 10th July 2020. If you will need more time than this to complete your revisions, please reply to this message or contact the journal office at plosone@plos.org. When you're ready to submit your revision, log on to https://www.editorialmanager.com/pone/ and select the 'Submissions Needing Revision' folder to locate your manuscript file.

Please include the following items when submitting your revised manuscript:

- A rebuttal letter that responds to each point raised by the academic editor and reviewer(s). You should upload this letter as a separate file labeled 'Response to Reviewers'.
- A marked-up copy of your manuscript that highlights changes made to the original version. You should upload this as a separate file labeled 'Revised Manuscript with Track Changes'.
- An unmarked version of your revised paper without tracked changes. You should upload this as a separate file labeled 'Manuscript'.

If you would like to make changes to your financial disclosure, please include your updated statement in your cover letter. Guidelines for resubmitting your figure files are available below the reviewer comments at the end of this letter.

If applicable, we recommend that you deposit your laboratory protocols in protocols.io to enhance the reproducibility of your results. Protocols.io assigns your protocol its own identifier (DOI) so that it can be cited independently in the future. For instructions see: <http://journals.plos.org/plosone/s/submission-guidelines#loc-laboratory-protocols>

We look forward to receiving your revised manuscript.

Kind regards,

Mayank Gururani
Academic Editor
PLOS ONE

**Dear Editor,**

**Thank you and the journal reviewers for useful comments on our manuscript. As described in detail below and also on the re-submitted manuscript, we have now revised the manuscript by carefully considering these comments.**

**We hope that the manuscript is now suitable for publication in PLOS ONE**

**Yours Sincerely**

**Prof. Dr. Ali ERGÜL**

**6. Review Comments to the Author**

Please use the space provided to explain your answers to the questions above. You may also include additional comments for the author, including concerns about dual publication, research ethics, or publication ethics. (Please upload your review as an attachment if it exceeds 20,000 characters)

**Reviewer #1:** The authors present a much improved manuscript. The manuscript provides a very thorough description of gene expression changes in grapevine leaves after TM and NaCl treatment. I believe the MS now conforms to PLoS ONE’s criteria for publication as my previous concerns about the fullness of the methods has now been addressed. I believe the MS could still be slightly improved by an explanation of tunicamycin in the introduction. Currently the first mention of tunicamycin is “These mutants accumulate misfolded proteins and induce the UPR in response to tunicamycin”. It would be useful to state something along the lines of “tunicamycin blocks glycoprotein synthesis and triggers the UPR” before this statement**.**

**Response: “In the revised manuscript, the statement has now been modified as per reviewer suggestion as ; Tunicamycin blocks glycoprotein synthesis and triggers the UPR. These mutants accumulate misfolded proteins and induce the UPR in response to tunicamycin.**

**Reviewer #2:** Comment to the Editor and Authors:
the text of the paper was improved. The figures have been changed and are impressive. Number of literature positions was shortened.
The number of paragraphs should be reduced more, by typing continuously without line breaks (in most cases). This is only the technical issue. Division into paragraphs should be done according to the main topic of particular text fragments
I recommend the paper for publication.
Krystyna Rybka

**Response: As per reviewer suggestion, the number of paragraphs has now reduced, by typing continuously without line breaks (in most cases). The division into paragraphs has been done according to the main topic of particular text fragments .**

**Reviewer #3:** The authors made all the recomended changes during the revision of the manuscript or they gave an appropriate answer for the remarks
